# A Randomized Control Trial of Three Intravenous Dexmedetomidine Doses for Procedural Sedation in Patients Undergoing Minor Gynaecological Surgery

**DOI:** 10.7759/cureus.23309

**Published:** 2022-03-19

**Authors:** Neha Sharma, Maitree Pandey, Anshu Gupta, Anil Kumar

**Affiliations:** 1 Anaesthesia and Critical Care, Lady Hardinge Medical College, New Delhi, IND; 2 Anaesthesiology, Lady Hardinge Medical College, New Delhi, IND; 3 Anesthesiology and Critical Care, Lady Hardinge Medical College, New Delhi, IND

**Keywords:** ramsay sedation score, modified aldrete score, postanesthesia discharge scoring system, gynaecological surgery, sedation, dexmedetomidine

## Abstract

Background: Minor gynaecological procedures are usually done in outpatient settings. Early discharge with minimal haemodynamic compromise is an essential requirement of these procedures. Many sedative drugs are being used for outpatient surgeries. Of the sedative agents available, dexmedetomidine, which has sedative and analgesic sparing effects, has the best safety profile in the cardiorespiratory system. Therefore, we evaluated the optimum dexmedetomidine dose for providing better procedural sedation.

Methodology: This randomized, double-blinded study included 120 ASA grade I and II patients aged 18-45 years who were undergoing short gynaecological procedures in a tertiary care hospital. Patients were randomly allocated into three groups of 40 each. After a loading dose of 1 µg/kg over 10 min, group A received dexmedetomidine infusion at a rate of 0.2 µg/kg/hr, group B at a rate of 0.4 µg/kg/hr, and group C at a rate of 0.6 µg/kg/hr. Perioperative hemodynamic changes, intraoperative adjuvant drug requirements, and postoperative recovery were also compared in the three different dexmedetomidine groups.

Results: Heart rate, blood pressure, oxygen saturation, and respiratory rate remained within the normal physiological range in all three groups at most perioperative time points. The time to achieve the Modified Aldrete Score and the post-anesthetic discharge scoring system was maximum in group C and minimum in group A. Ketamine had to be supplemented in almost half of the patients in group A and less than a quarter of the patients in group B. In group C, surgery was completed without any drug supplementation. Two patients in group B and four patients in group C had an episode of bradycardia. Oxygen saturation decreased in one patient in group C, necessitating oxygen supplementation.

Conclusions: Dexmedetomidine, at a dose of 0.4 µg/kg/hr with ketamine supplementation, provides the most appropriate procedural sedation and analgesia (PSA) without any significant hemodynamic compromise.

## Introduction

Minor surgery, despite its short duration, is associated with significant pain and discomfort. Several anesthetic techniques and pharmacological agents have been used to reduce patient discomfort and facilitate surgical performance. However, procedural sedation and analgesia (PSA) is still preferred over general anesthesia during short gynaecological procedures [1].

Most analgesics/sedative drugs, such as midazolam, propofol, and fentanyl, which are commonly used for PSA, can potentially prolong sedation and cause respiratory depression and adverse hemodynamic effects, which may result in increased morbidity and unplanned hospitalization as most cases are done as daycare surgery [2].

Dexmedetomidine, a known sedative and analgesic sparing drug that acts on α2 adrenoceptor, reduces heart rate, blood pressure, and anesthetic drug requirements in response to any stress [3,4]. Unlike other anesthetics, it also has a sedative response, mimicking natural sleep without significant respiratory depression [5].

Dexmedetomidine is extensively used as a sedative and analgesic agent in various surgeries, but the optimum dose of dexmedetomidine in these procedures is still unknown. Thus, we designed this study to evaluate the optimum dexmedetomidine dose for short gynecological surgery. Our secondary objectives were to observe perioperative hemodynamic changes and any additional intraoperative adjuvant drug requirements, as well as to evaluate postoperative parameters using the Modified Aldrete Score.

## Materials and methods

**Figure 1:**
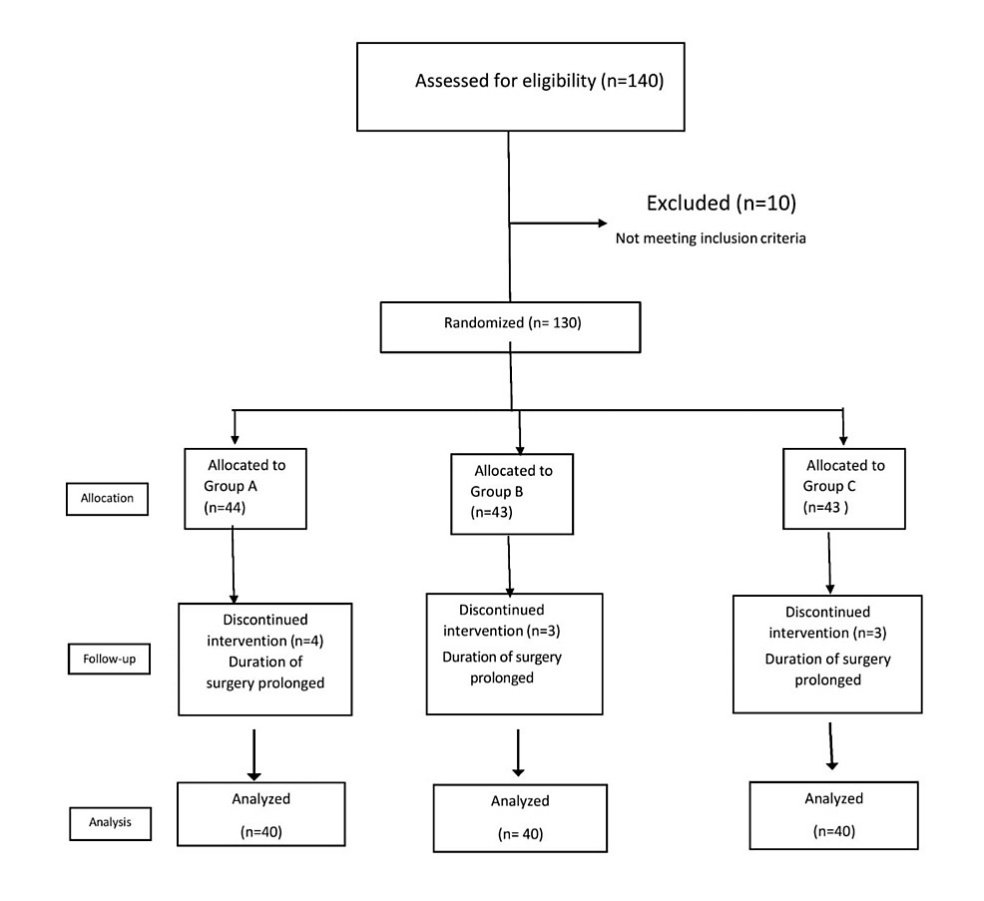
Consort flow diagram

The study was a randomized control trial conducted in a tertiary care hospital after approval by the concerned Institutional Ethical Committee (LHMC/ECHR/2014/27). The study population consisted of ASA grade I and II patients aged 18-45 years who were scheduled to undergo short gynecological surgery (20-40 min) under intravenous sedation and analgesia. We included hysteroscopic copper T removal, dilatation and curettage, hysteroscopic biopsy, and Bartholin cyst excision in the short gynecological surgery. We excluded patients with hypertension, cardiopulmonary diseases, hepatic disease, or who were allergic to any drug from the study. Assuming a 30 min difference in sedative effect between the two groups based on the pilot study with α = 0.05 and an 80% power, the minimum number of cases required under each group was 40, making it a total of 120 patients in three groups (Figure 1).

A computer-generated random table did randomization into three groups: (i) group A: dexmedetomidine infusion @ 0.2 µg/kg/hr; (ii) group B: dexmedetomidine infusion @ 0.4 µg/kg/hr; group C: dexmedetomidine infusion @ 0.6 µg/kg/hr.

The different doses of dexmedetomidine infusion were prepared by medical personnel not involved in the study. We prepared infusion drugs in a 50-ml syringe, and concentration varied in other groups. In groups A, B, and C, 100 µg, 200 µg, and 300 µg of dexmedetomidine were added in a 50-ml syringe, respectively. After a detailed preanesthetic checkup and investigations, patients' informed consents were taken for monitored anesthesia care, surgery, study participation, and publication.

Patients fasted for eight hours before the procedure. An intravenous catheter was placed in the preoperative room, and 50-mg intravenous ranitidine and 4-mg ondansetron were administered 30 min before the start of surgery. On arrival in the operation room, standard monitors were attached and baseline readings were recorded. Ringer lactate was started at 4 ml/kg/hr, and fentanyl was administered at 1 µg/kg before the procedure. All three groups received a loading dose of 1 µg/kg/hr of dexmedetomidine over the initial 10 min. After 10 min, a paracervical block was provided with 10 ml of 1.5% lignocaine by an experienced gynaecologist, and dexmedetomidine infusion was started at 0.1 ml/kg/hr in all three groups. The person monitoring the patient was blinded to the doses of dexmedetomidine being used for infusion. Injection Ketamine (0.3 mg/kg) was given as rescue analgesia if the patient complained of pain or refused surgery at any time point. Dexmedetomidine infusion was stopped at the end of the surgery, and the patient was transferred to the post-anesthesia care unit (PACU). Ramsay Sedation Score was assessed after shifting the patient to PACU [6].

Heart rate, systolic blood pressure, diastolic blood pressure, mean blood pressure, peripheral oxygen saturation, and respiratory rate were monitored at the baseline, every 5 min thereafter until surgery was completed, and every 15 min for the first hour followed by every 30 min until the patient was discharged. Complications (both intra-operative and post-operative) were recorded and treated accordingly. Recovery was assessed using the Modified Aldrete Score, and the time taken to reach Modified Aldrete Score ≥8 was noted. Thereafter, the patient was transferred to the post-operative ward [7]. The time taken to achieve Post-anaesthetic Discharge Scoring System (PADSS) ≥9 was recorded as readiness for discharge [8].

Statistical analyses

The data was compiled, tabulated, and statistically analyzed using Statistical Product and Service Solutions (SPSS) version 17 (IBM Corp., Armonk, NY). Analysis of variance was used for the analysis of mean difference among groups and Chi-square test for grading of sedation, recovery, and discharge. Results were presented as mean ± SD. Probability values less than 0.05 were considered significant.

## Results

All three groups had comparable demographic profiles (Table 1).

**Table 1:** Demographic profile Data are expressed as mean ± SD

	Group A (n=40)	Group B (n=40)	Group C (n=40)	P-value		
				A vs B	A vs C	B vs C
Age (years)	34.78 ± 7.54	34.50 ± 7.39	34.28 ± 7.14	0.435	0.381	0.445
Weight (kg)	56.78 ± 7.97	56.00 ± 6.51	55.8 ± 6.68	0.318	0.278	0.448

All patients had a Ramsay Sedation Score of 3 or more. In half of the patients in group A and in one-fourth of the patients in group B, ketamine was required as a rescue drug. None of the patients in group C required any drug supplementation (Table 2).

**Table 2:** Sedation score and supplementary drug requirements

Parameters	Group A		Group B		Group C	
	Freq. (n)	%	Freq. (n)	%	Freq. (n)	%
Sedation score						
3	17	42.5	18	45	13	32.50
4	23	57.5	22	55	27	67.50
Ketamine supplementation	18	45	9	22.5	0	0

In group A, the time taken to achieve the Modified Aldrete Score (≥8) was 21.14 ± 9.99 min and was almost doubled in groups B (39.68 ± 18.39 min) and C (45.38 ± 29.90 min). This difference was statistically significant in the A versus B group and the A versus C group (p = 0.000). However, it was comparable between groups B and C (p > 0.05) as shown in Table 3. Patients in group A achieved PADSS score (≥9) earlier (148.64 ± 23.56 min) than in group B (177.10 ± 16.16 min) and group C (200.25 ± 18.47 min). This time, the difference in the achievement of the PADSS score was statistically significant among all three groups (p < 0.001), as shown in Table 3.

**Table 3:** Data are expressed as mean ± SD *Signifies p-value < 0.05

Parameters	Group A	Group B	Group C	p-value		
				Avs B	A vs C	B vs C
Time to achieve Modified Aldrete Score	21.14 ± 9.99	39.68 ± 18.39	45.38 ± 29.90	0.000^*^	0.000^*^	0.177
Time to achieve PADSS Score	148.64 ± 23.56	177.10 ± 16.16	200.25 ± 18.47	0.000^*^	0.000^*^	0.000^*^

**Figure 2:**
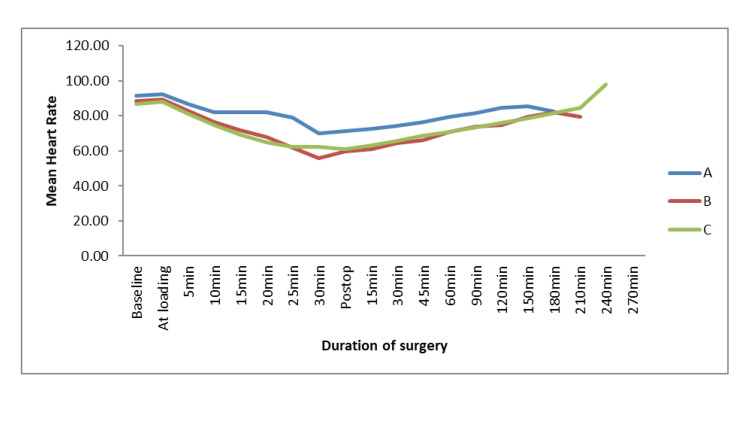
Intra-operative and post-operative mean heart rate

The mean heart rate decreased significantly (p < 0.05) in all three groups (Figure 2), whereas the baseline heart rate was comparable in all groups. After that, the difference in heart rate was highly significant between A versus B and A versus C. However, groups B and C were comparable. All three groups showed a gradual increase in heart rate during the post-operative period. There was no significant difference in heart rate among all groups at discharge.

**Figure 3:**
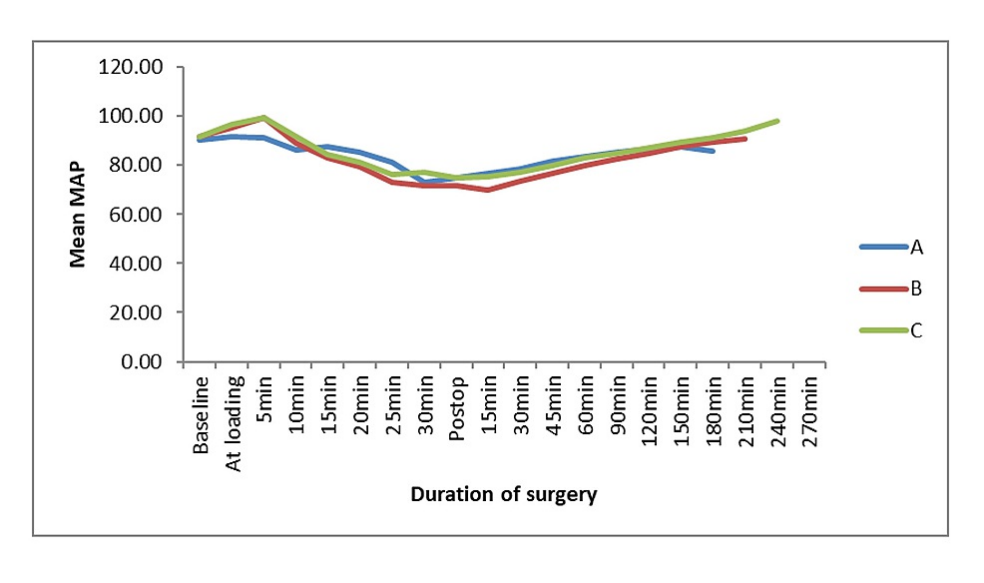
Intra-operative and post-operative mean arterial pressure

At baseline, blood pressure was comparable in all three groups (Figure 3). With the start of dexmedetomidine infusion, there was a transient increase in blood pressure in all the groups, followed by a gradual decrease. Although the decrease in blood pressure was statistically significant, it was clinically insignificant. Blood pressure remains comparable in groups B and C at most time points. Post-operative blood pressure followed a rising trend to reach a baseline value at the time of discharge in all three groups.

**Figure 4:**
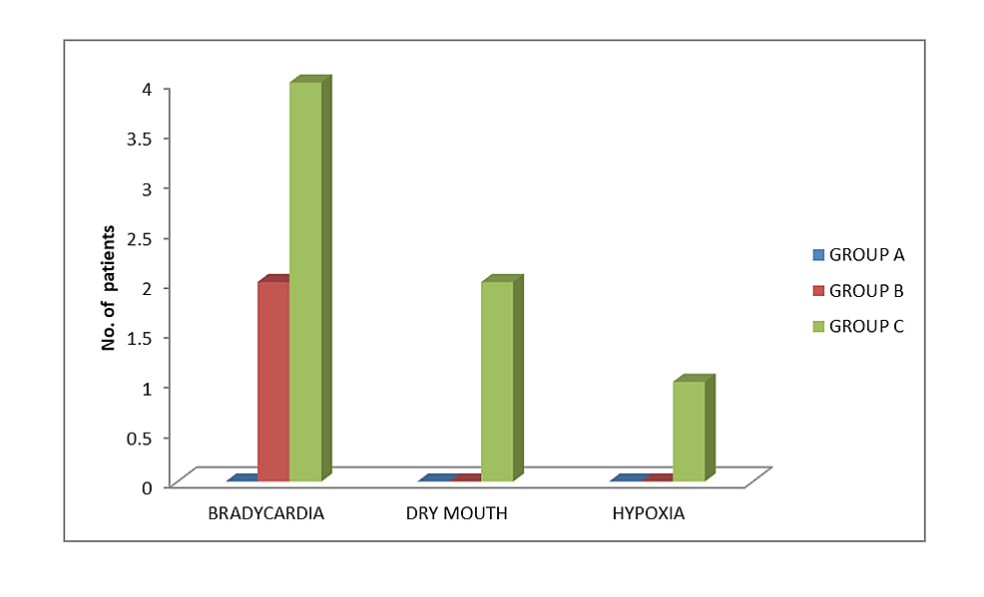
Side effects

Throughout the perioperative period, at no time did the mean oxygen saturation decrease below 94% in any of the three groups, except for one patient in group C at 20 min after starting the loading dose, which was improved with oxygen administration via the face mask. Mean oxygen saturation increased postoperatively and remained within the normal physiological range (Figure 4). The respiratory rate remained within the normal physiological range both intra and postoperatively in all three groups (Figure 4). Bradycardia was observed in two patients in group B and four patients in group C, which was corrected by injection of atropine. Dry mouth was observed in two patients in group C. Out of 120 patients, only one patient in group C had to be supplemented with oxygen (SpO_2_ < 92%).

## Discussion

This study was a prospective, randomized, double-blinded control trial conducted in patients undergoing minor gynecological surgeries to determine the optimum dose of dexmedetomidine infusion for procedural sedation in such surgeries. Out of 140 patients who were assessed for this study, only 130 met the inclusion criteria. Although 10 patients were eliminated from the study due to prolonged surgical duration (more than 40 minutes), we continued procedural sedation and analgesia in these patients.

The use of general anesthesia or central neuraxial blocks during short gynecological procedures is inappropriate. These techniques not only delay the discharge but also increase morbidity. PSA, which combines the use of local anesthesia with intravenous sedation, can be the technique of choice during such short procedures.

Midazolam, propofol, and fentanyl are the most commonly used medications for PSA. Combining midazolam with fentanyl or propofol increases the risk of hypoxemia and apnea [9]. However, due to its minimal effect on haemodynamic variables and conscious sedation that imitates natural sleep, dexmedetomidine may be considered an ideal sedative agent.

Our study discovered that hemodynamic and respiratory variables were maintained within physiological limits during most time points in all three groups. Sedation scores of 3 or higher were achieved by a nearly equal number of patients in groups A and B. This contradicted the findings of Angst et al., who discovered that sedation scores increased in a plasma concentration-dependent manner [10]. This difference could be explained by the fact that a greater number of patients in group A required ketamine supplementation intraoperatively compared to group B, which increased the sedation score of group A. Similar to this study, several authors have documented optimum sedation with a dexmedetomidine infusion rate of 0.2 µg/kg/hr [11-13]. A sedation score of >3 was observed when a 0.6 µg/kg/hr infusion dose was used, which was similar to group C of our study [14].

The need for supplementary analgesic drugs decreased with an increase in the dose of dexmedetomidine infusion, with no patients in group C requiring any ketamine supplementation. Similar to our results, Aho et al. observed that only 33% of patients who received 0.4 µg/kg/hr dexmedetomidine required analgesic supplementation [15]. While Parikh et al. observed only 11% of patients required rescue analgesia when 0.2 µg/kg/hr of dexmedetomidine infusion was used [12]. This was much less as compared to group A of this study.

The time taken to achieve a Modified Aldrete Score ≥8 and a Postanesthetic Discharge Scoring system (PADSS) >9 dose-dependently increased and reached its maximum with higher doses of dexmedetomidine infusion. A similar recovery time was observed in other studies [16,17]. However, recovery time similar to group C was observed with a dose of 0.2 µg/kg/hr in a study [18]. This may be due to the fact that their recovery criteria were different and the procedure was less painful.

In a study, readiness to discharge was observed to be 97.3 ± 7.7 min, with a dexmedetomidine infusion rate of 0.2 µg/kg/hr, and it was much less than group A of our study [13]. This difference may be due to the higher loading dose of dexmedetomidine and the use of ketamine in a greater number of patients in our study.

The mean heart rate began to decrease 5 min after the start of dexmedetomidine infusion in all three groups and continued till the end of surgery. This decline in heart rate was statistically significant (p < 0.05) in all three groups. According to previous studies, different doses of dexmedetomidine infusion exhibited a similar trend [11,12,14,19-22].

There was a gradual increase in heart rate during the postoperative period, and it approached baseline values at the time of discharge of patients in all three groups. Contrary to this, a few studies have observed a significant decrease in heart rate even after 60 min of post-infusion with 0.2 µg/kg/hr and 0.4 µg/kg/hr doses [11,12,20]. There was no significant intergroup difference in terms of heart rate [20].

There was a brief, clinically insignificant increase in mean blood pressure after the start of loading drug infusion in all three groups, followed by a gradual decrease until 30 min of dexmedetomidine infusion. This brief increase can be explained by the fact that peripheral α2b receptors are activated earlier compared to central receptors [11,20]. A decrease in mean blood pressure was also observed in other studies with different concentrations of dexmedetomidine infusion [11,12,14,19-22]. Mean blood pressure gradually increased after the stoppage of the infusion and returned to baseline values in all three groups.

In our study, the incidence of bradycardia was 5% in group B and 10% in group C, but Park et al. reported a lower incidence of bradycardia compared to our study [23]. The higher incidence of bradycardia in our study could be due to the additive effect of the injection of fentanyl administered.

After the start of loading of the study drug, all groups experienced a statistically significant but clinically insignificant decrease in oxygen saturation. Similar to the study of Eren et al., only one patient in group C required oxygen supplementation [24].

In group C, two patients (5%) developed dryness of the mouth. This is consistent with the results of another study [11]. No patients in any group required postoperative supplementation for pain.

## Conclusions

Dexmedetomidine at a dose of 0.6 µg/kg/hr provides efficient sedation and analgesia but is associated with significant hemodynamic compromise, whereas dexmedetomidine at a dose of 0.4 µg/kg/hr requires ketamine supplementation at 0.3 mg/kg to achieve adequate analgesia and sedation without hemodynamic complications. Hence, we recommend using ketamine with low-dose dexmedetomidine (0.4 µg/kg/hr) for procedural sedation in minor gynecological surgeries. This study has a few limitations, such as we had not observed the effect of dexmedetomidine in male patients and the population size was small. More studies with a larger population are required to affirm our conclusion.
